# Specific coping strategies moderate the link between emotion expression deficits and nonsuicidal self-injury in an inpatient sample of adolescents

**DOI:** 10.1186/s13034-017-0158-3

**Published:** 2017-04-13

**Authors:** Kristel Thomassin, Camille Guérin Marion, Myriam Venasse, Anne Shaffer

**Affiliations:** 1 School of Psychology, University of Ottawa, 136 Jean Jacques Lussier, Ottawa, ON K1N 6N5 Canada; 2 Department of Psychology, University of Georgia, 300 Hooper Street, Athens, GA 30606 USA

**Keywords:** Nonsuicidal self-injury, Adolescent, Coping

## Abstract

**Background:**

Non-suicidal self-injury (NSSI) is a behavior of increasing prevalence in adolescents with links to various negative mental health and adjustment outcomes. Poor emotion expression has been linked with NSSI use, whereas the use of adaptive coping strategies has been identified as a protective factor against NSSI. The current study examined whether specific coping strategies moderate the relation between poor emotion expression and NSSI, and whether moderation is conditional on adolescent gender.

**Methods:**

Ninety-five adolescents hospitalized on an acute care inpatient psychiatric unit completed questionnaires measuring NSSI, emotion expression and use of specific coping strategies (i.e., problem-focused coping, positive reframing coping, support seeking, avoidance, and distraction).

**Results:**

Results indicated that poor emotion expression was positively associated with NSSI. Positive reframing and support seeking emerged as significant moderators of the poor emotion expression—NSSI link. This result was not conditional upon adolescent gender. Problem-focused coping, avoidance, and distraction did not emerge as significant moderators.

**Conclusions:**

Encouraging youth to use particular coping strategies might protect against the negative impact of emotion expression deficits for both boys and girls.

## Background

Nonsuicidal self-injury (NSSI) is the intended destruction or alteration of one’s body tissue without conscious intention to attempt suicide [1]; NSSI includes behaviors that are not culturally normative and manifests most commonly as cutting, burning, scratching, and head banging/hitting, among other methods [2]. Particularly alarming is the commonplace occurrence of NSSI within psychiatric adolescent samples, with reported rates ranging from 40% to as high as 82% [3, 4]. In light of this, much research has focused on correlates as well as risk and protective factors of NSSI among youth. At the foreground of this research has been the role of emotion-related deficits in adolescents who engage in NSSI [3, 5]. In particular, previous work has focused on dysregulated emotional expression, including poor emotion awareness and reluctance to express emotions, as key factors associated with NSSI [6–10]. It is thus important to consider factors that might buffer the negative impact of emotion expression deficits on NSSI. The current study examined whether specific coping strategies moderate the link between poor emotion expression (i.e., poor emotion awareness and reluctance to express emotion) and NSSI in an inpatient adolescent sample.

### Emotion expressivity and NSSI

Consistent with theoretical models of emotional disturbances [7] and NSSI including Linehan’s Biosocial Model [5, 6] and Yates’ Developmental Psychopathology model of NSSI [8], a majority of adolescents report engaging in NSSI to obtain rapid—albeit temporary—relief from intense negative emotions [5, 6]. Accordingly, considerable research has accumulated on the links between emotion deficits and NSSI in adolescents. Poor emotion expression in particular warrants attention as a key risk factor for NSSI. For instance, Gratz [9] found that low emotion expression was associated with more frequent self-harm among young college females. In another study, Sim and colleagues [10] found that emotion expression mediated the link between parental reactions to children’s display of emotions and NSSI in an inpatient sample of adolescent girls. These emotion expression skills—emotion awareness and willingness to express—have been identified as correlates and predictors of NSSI in past research [10–14]. Individuals who have difficulty with emotion awareness are likely to also have difficulties modulating their emotional responses to stimuli, primarily because they cannot match emotion regulation strategies to the emotion experienced [10]. Regarding emotion expression, a robust body of literature suggests that emotional suppression leads to paradoxical effects such as increases in sympathetic nervous system activity [15]. Further, adolescents who are unwilling to express their emotions might not have access to support from others as a means of regulating distress. Access to useful coping strategies might be particularly relevant to these adolescents. Given these findings, it is important to consider factors that might mitigate the impact of poor emotion expression on NSSI. In the current study, we investigated unique coping strategies as potential mitigating factors.

### Coping and NSSI

Coping is the intentional and deliberate efforts used to manage emotions and/or situations that pose a threat to the individual. These efforts may or may not be emotion-focused and may lead to resolution of the problem, or to accommodation of the concern without a solution [16]. Previous research has identified differences in coping among adolescents who engage in NSSI versus those who do not [17, 18]. Guerreiro and colleagues [19] reviewed the extant literature on coping and adolescent self-injury from 2000 to 2010 and concluded that coping strategies generally deemed “adaptive” (e.g., problem-focused coping, positive reframing, support seeking) are consistently associated with a lower risk of NSSI in adolescents, whereas “maladaptive” coping strategies (e.g., avoidance) are generally linked with higher rates of NSSI [19], although it should be noted that the review did not differentiate suicide attempts from NSSI. Additionally, some mixed findings suggest these links likely differ based on context (e.g., distraction has also proven helpful for self-harmers; [18]). Another study by Santos et al. [20] found that youth aged 15–24 years who reported a history of NSSI were less likely than youth without a history of NSSI (matched by age, gender and residence) to engage in support seeking and problem-focused coping in the face of problems (i.e., in situations posing threat, harm or a challenge) [20]. It should be noted that the authors did not differentiate individuals with and without suicidal intent. Nonetheless, research has shown that adaptive coping is associated with a reduced likelihood of engaging in NSSI, both in the presence and absence of past suicide attempts [18]. Research has also identified links between coping and severity of NSSI. For example, Voon et al. [21] found that adolescents who engaged in cognitive reappraisal exhibited reductions in the severity of the NSSI incidents over time, controlling for psychological distress, adverse life events, and suicide attempt history. Given that coping is implicated in NSSI, it is important to test whether specific coping strategies could be protective for adolescents with emotion expression deficits.

In terms of coping as a potential protective factor in predicting NSSI, a study conducted by Williams and Hasking [22] found that emotion-focused coping and avoidant coping moderated the relation between psychological distress and NSSI in young adults. While this study points to an interaction between distress and coping in predicting NSSI, to our knowledge, no research has tested the moderating role of specific coping strategies in the link between emotion expression deficits and NSSI. This is important given the robust link between poor emotion expression and NSSI. Discomfort with or inability to express emotions could be less detrimental in contexts where adolescents are able to use effective coping strategies to respond to distressing emotions in other ways.

### The current study

The current study examined the moderating role of five specific coping strategies (problem-focused coping, support seeking, positive reframing, avoidance, and distraction) on the relation between poor emotion expression and NSSI in a sample of adolescents hospitalized on an inpatient unit. Based on previous research, it was hypothesized that poor emotion expression would be positively linked with adolescent NSSI and that engaging in adaptive coping strategies (i.e., problem-focused, positive reframing, distraction, and support seeking) would buffer the impact of poor emotion expression on NSSI. Exploratory analyses tested adolescent gender as an additional moderator given previous research indicating gender differences in emotion expressivity [23], NSSI methods and motives [24, 25], as well as in coping strategy use [26].

## Methods

### Participants

A total of 95 adolescents between the ages of 10–17 years (*M* = 14.22, *SD* = 1.67, 58% girls) were recruited from two psychiatric hospital inpatient units located in the Southeastern United States. The ethnic breakdown of the sample of participants was Caucasian (56%), African American (35%), Hispanic (3%), Asian (1%), and other (3%). Two percent of the sample did not report on ethnicity. The past medical records of all participants were examined to determine the presence of any primary psychological diagnoses. Primary diagnoses included depression or mood disorders (52%), externalizing disorders (i.e. ODD, conduct disorder or ADHD; 13%), posttraumatic stress disorder or other anxiety disorders (11%), bipolar disorder (10%), psychosis or schizophrenia (4%), and other diagnoses (i.e., substance abuse and gender identity disorder; 4%). There was no primary diagnostic information available for 2% of the sample, and 4% of participants had no primary diagnosis. Psychiatric comorbidity was also present in 63% of participants.

### Measures

#### Nonsuicidal self-injury

The *Deliberate Self*-*Harm Inventory* (DSHI; Adapted from Gratz [27]) asked adolescents to report on their frequency of NSSI for 17 different self-harm behaviors on a multiple choice scale from (a) 1–2, (b) 3–5, (c) 6–12, and (d) more than 12 times. NSSI methods included: cutting, punching self, burning skin with lighter or match, carving words or designs into skin, scratching self, biting self, rubbing sandpaper on skin, dripping acid onto skin, using bleach to scrub skin, sticking sharp objects into skin, rubbing glass into skin, breaking one’s own bones, head banging, preventing wounds from healing, and “other”. For each NSSI behavior, respondents were also asked a subset of specific questions on the nature of their use of the method (e.g., age of onset, last use of the method). Given that previous research has questioned the inclusion of “preventing wounds from healing” [28–30], we omitted this item from the total score. The total lifetime NSSI frequency was calculated from the sum of the frequencies for each individual method endorsed by the adolescent (α = .85).

#### Emotion expression

The *Emotion Expression Scale for Children* (EESC; [31]) is a self-report measure composed of 16 items that aims to assess youth’s awareness of emotions and willingness to communicate emotions to others. Youth are asked to respond to items using a 5-point Likert scale with anchors ranging from 1 (*not at all true*) to 5 (*extremely true*), which yield two subscales: poor emotion awareness (e.g., “I often do not know why I am angry”, or “I have feelings that I can’t figure out”) and expressive reluctance (e.g., “I prefer to keep my feelings to myself”, or “When I’m sad, I try not to show it”). In the current study, the two subscales were highly correlated (*r* = .75, *p* < .001) and were therefore averaged into a composite score (α = .90), with higher scores reflecting poorer emotion expression.

#### Coping

The *Children’s Coping Strategies Checklist* (CCSC; [32]) is a 54-item self-report measure, which yields five broadband subscales: problem-focused coping (12 items; e.g., “you thought about which things are best to do to handle the problem”), support seeking (9 items; e.g., “you talked to someone who could help you figure out what to do”), positive reframing (12 items; e.g., “you tried to notice or think about only the good things in your life”), avoidance (12 items; e.g., “you tried to ignore it”), and distraction (9 items; e.g., “you went bicycle riding”). Participants are asked to rate items on a 4-point Likert scale ranging from 1 (*never*) to 4 (*most of the time*). Cronbach’s alphas ranged from acceptable to good (problem-focused coping, α = .88; positive reframing coping, α = .89; support seeking, α = .88; avoidance, α = .81; distraction, α = .56).

#### Psychopathology symptoms

Symptoms of psychopathology were assessed via the Youth Self-Report [33]. The Youth Self-Report is a 113-item self-report questionnaire that assesses for broadband internalizing and externalizing symptoms. The measure exhibits very strong psychometric properties [33]. For the current study, the total problems subscale was used; internal consistency was .96.

### Procedure

Adolescents’ legal guardians provided informed consent at admission to the psychiatric units. Unit staff met with adolescents to obtain assent, and research staff met with each adolescent individually to administer the questionnaires. As an incentive for participation, adolescents were provided tokens, which they could claim at an on-site token store. All procedures were conducted in accordance with the Institutional Review Boards of the sponsoring University and hospitals.

### Data analytic plan

Analyses first examined means, standard deviations, and intercorrelations among all study variables. Rates of NSSI and coping were examined for the whole sample and separately by adolescent gender. Five moderation models, one for each of the coping strategies previously mentioned (i.e., problem-focused coping, positive reframing coping, support seeking, avoidance, and distraction), were tested using MPlus 7.0 [34]. A zero-inflated poisson regression analysis was used, which treated the NSSI frequency variable as a count variable. This approach was used to account for the fact that NSSI frequency was highly and positively skewed in our sample. Bootstrapping techniques (1000 resamples) were also employed as a robust technique to reduce bias due to potential non-normality [35]. Models were run using full information maximum likelihood (FIML; [34]) to account for missing data, which ranged from 0 to 2.2%. All models included adolescent age and gender as covariates and explored adolescent gender as an additional moderator. However, adolescent gender was not a significant moderator in any of the models. Therefore, model testing interpreted the 2-way interaction between poor emotion expression and coping strategy. If significant, the interaction between poor emotion expression and coping was examined at low and high levels of the moderator variable (i.e. ∓1 *SD* below/above the mean). All significant interactions were graphed to facilitate interpretation.

## Results

### Descriptive analyses

Initial analyses first examined whether participants from the two sites differed on any of the variables examined in the current study. Results indicated that adolescents differed across sites in terms of age, *F* (1, 93) = 14.46, p < .001. Even though age was not correlated with other study variables, we included age as a covariate in all moderation models to account for these differences. In terms of descriptive details regarding levels of NSSI in the current sample, just over two-thirds of adolescents reported a history of NSSI (72%, *n* = 68), with approximately 84% of girls and 55% of boys endorsing NSSI. The gender difference in frequency of NSSI was not significant, *F* (1, 94) = .03, *p* = .863. Of the 17 methods in the DSHI, the most prevalent behaviors in girls were cutting (76%), sticking sharp objects into skin (76%), and scratching (35%). Boys with a history of NSSI most frequently reported engaging in head banging (35%), cutting (33%), and burning with a lighter or match (28%). Psychopathology symptoms were not associated with NSSI frequency, so these were not included as a covariate in further analyses. In regard to coping strategies, there were no significant gender differences in coping strategies endorsed when the whole sample was examined. When examining the subset of adolescents who engaged in NSSI, adolescent girls reported engaging in significantly more avoidance than boys, *F* (1, 64) = 4.34, *p* = .041. One-way ANOVAs also tested whether coping strategies differed between adolescents who had engaged in NSSI versus those who had not, and no significant differences emerged. Poor emotion expression was significantly and positively correlated with NSSI frequency (*r* = .38*, p* < .001) (Table 1).

**Table 1:** Means, standard deviations, and intercorrelations among all study variables

Variable	2	3	4	5	6	7	M_girls_ (*SD*)	M_boys_ (*SD*)
1. Nonsuicidal self-injury	.38***	−.13	−.20	−.25*	−.08	−.05	17.80 (21.67)	17.80 (34.66)
2. Poor emotion expression	–	.14	.12	−.08	.39***	.14	26.58 (6.47)	23.38 (7.30)
3. Problem-focused		–	.77***	.61***	.60***	.49***	2.36 (.63)	2.34 (.78)
4. Positive reframing			–	.65***	.64***	.51***	2.16 (.64)	2.35 (.77)
5. Support seeking				–	.43***	.39***	2.02 (.73)	2.20 (.78)
6. Avoidance					–	.35**	2.67 (.55)	2.45 (.67)
7. Distraction						–	2.40 (.73)	2.49 (.69)

* *p* < .05, ** *p* < .01, *** *p* < .001

### The moderating role of coping and gender on the link between poor emotion expression and NSSI

**Fig. 1:**
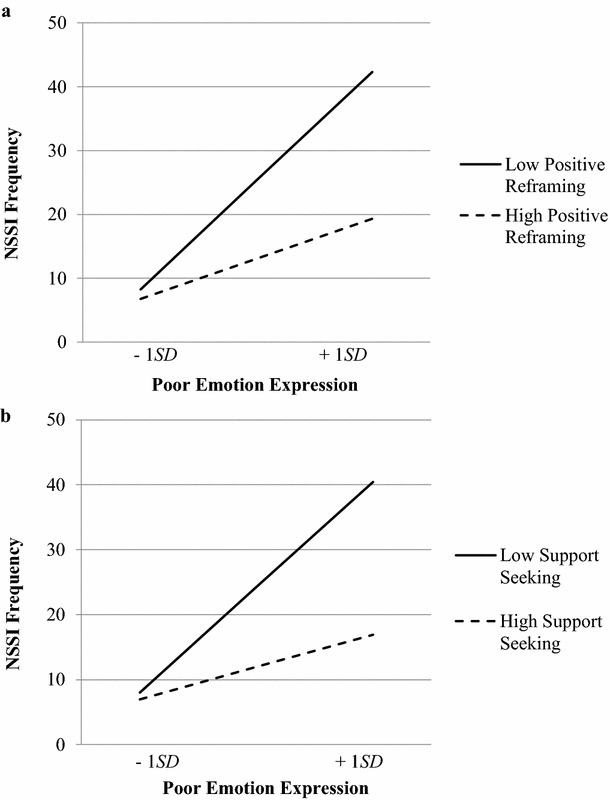
Conditional effects of emotion expression on NSSI at low (−1*SD*) and high levels (+1*SD*) of positive reframing (**a**) and support seeking (**b**)

Problem-focused coping, distraction, and avoidance did not moderate the link between poor emotion expression and NSSI, *b* = −.13, *p* = .945, 95% CI [−4.28, 2.98], *b* = −1.52, *p* = .368, 95% CI [−4.85, 1.71], and *b* = −2.29, *p* = .124, 95% CI [−5.04, .59], respectively. In the moderation model examining problem-focused coping, none of the main effects were significant, including the main effect of poor emotion expression, *b* = 1.13, *p* = .223. In the models examining distraction and avoidance, the main effects of poor emotion expression were significant, *b* = 2.09, *p* = .036, and *b* = 2.07, *p* = .010, respectively. In terms of positive reframing and support seeking, both moderated the emotion expression-to-NSSI association, and these are described below. Graphed interactions are presented in Fig. 1.

The 3-way interaction between poor emotion expression, positive reframing coping, and gender was not significant, *b* = 1.07, 95% CI [−.22, 2.54], but the 2-way interaction was significant, *b* = −3.08, 95% CI [−5.35, −.55]. The interaction was then probed at low (−1 *SD* below the mean) and high (+1 *SD* above the mean) levels of positive reframing. At low levels of positive reframing, poor emotion expression was positively associated with NSSI, *b* = .11, 95% CI [.02, .19], but at high levels of positive reframing, this link was no longer significant, *b* = .03, 95% CI [−.03, .12]. These betas at low and high levels of positive reframing coping were significantly different from one another, t(37) = 6.81, *p* < .001.

In the moderation model examining support seeking, the 3-way interaction between poor emotion expression, support seeking and gender was not significant, *b* = .21, 95% CI [−1.06, 1.42]. However, the 2-way interaction between poor emotion expression and support seeking was significant, *b* = −2.00, 95% CI [−4.30, −.13]. At low levels of support seeking (−1 *SD* below the mean), poor emotion expression was positively associated with NSSI, *b* = .11, 95% CI [.04, .19]; at high levels of support seeking (+1 *SD* above the mean), there was no significant association between poor emotion expression and NSSI, *b* = .04, 95% CI [−.20, .10]. These betas at low and high levels of support seeking were significantly different from one another, t(51) = 6.94, *p* < .001.

## Discussion

Given robust links between emotion deficits and NSSI [10–14], the current study built on the extant literature by examining the moderating role of specific coping strategies in the links between poor emotion expression (i.e., poor emotion awareness and reluctance to expression emotion) and NSSI. It was expected that adaptive coping strategies would mitigate the negative impact of emotion deficits on NSSI. Overall, results supported positive reframing and support seeking as significant moderators of the poor emotion expression-to-NSSI link, regardless of adolescent gender. Problem-focused coping, distraction, and avoidance did not emerge as significant moderators.

In terms of our sample characteristics, the prevalence rate of NSSI in the current sample was 71.6%, which is consistent with previous research with adolescent inpatient samples (i.e., 40–82%; [3, 4]). Results regarding the methods used by adolescent girls and boys were also consistent with previous research [25], with cutting being the most common NSSI method in girls (76%) and head-banging being the most common for boys (35%). As expected, poor emotion expression was positively linked with NSSI frequency. It is likely that adolescents who have difficulties identifying and communicating their internal emotional states are less able to implement adaptive regulation strategies when faced with intense negative emotion, thus increasing the likelihood of them resorting to short-term and impulsive strategies such as NSSI to relieve negative affect [10, 36, 37]. This result also corroborates past research findings [10, 12] and theory [37] suggesting that NSSI may serve a regulatory function when youth are unable or reluctant to express emotions.

Regarding coping strategies, the use of various strategies did not differ significantly between NSSI and non-NSSI individuals or by gender. As expected, engaging in positive reframing appeared to buffer the negative impact of poor emotion expression on adolescent NSSI. In other words, at low levels of positive reframing, poor emotion awareness and reluctance to express emotion were positively associated with NSSI, whereas at high levels of positive reframing coping, these links were no longer significant. This finding aligns with research that indicates that NSSI severity is reduced in adolescents who report greater use of cognitive restructuring, a coping strategy similar to positive reframing [21]. The application of these findings could prove valuable in the context of cognitive restructuring exercises in cognitive-behavioral therapy, where an emphasis on positive reframing could enhance therapeutic effects for adolescents with emotion deficits and NSSI histories.

Support seeking also emerged as a significant moderator, which is consistent with past research showing that adolescents who do not engage in self-injurious behaviours—whether driven by suicidal intent or not—have a higher tendency to use strategies such as talking to someone to attempt to cope with stressors [38]. Specifically, within the context of poor emotion expression, support seeking may encourage adolescents to process their emotional experience through dialogue, thereby supporting introspective thinking and helping them become more cognizant of how they feel. Support seeking may also be helpful particularly if the support can be validating to the adolescent or can offer regulatory help. This may in turn decrease the adolescent’s urge to turn to NSSI as a regulatory strategy [37].

Interactions between coping strategy and emotion expressivity problems did not differ by gender, suggesting that these coping strategies can be effective for adolescent boys and girls. In particular, this pattern of results appears to override gendered coping norms, which generally reinforce the value of seeking support (i.e., showing vulnerability, expressing emotions) for girls to a greater extent than for boys.

Problem-focused coping and distraction were not significant moderators. This was unexpected given previous research suggesting these coping strategies are associated with lower odds of engaging in NSSI (e.g., [18, 38]). It could be that these particular strategies are not as helpful in the context of emotion expression deficits. Problem-focused and distraction approaches might be too challenging for severely dysregulated adolescents to implement in the moment in order to not engage in NSSI. As expected, avoidance was not a significant moderator. Because the extant literature indicates avoidance is associated with greater levels of NSSI [19], we did not expect avoidance to protect adolescents from the negative impact of poor emotion expression on NSSI.

## Limitations and future directions

The current study is not without limitations. The cross-sectional design does not allow causal inferences among study variables, and the sample did not allow for the inclusion of various confounding variables such as current psychiatric diagnoses, previous suicide attempts, and number of previous admissions. The assessment of previous psychiatric diagnoses was conducted via file review and was therefore not corroborated by a second reviewer. The sample was moderate in size and constructs were assessed via self-report. The current study’s assessment of emotion expression was based on adolescents’ own evaluation of their emotion awareness and willingness to express emotion. It would be informative to incorporate additional ways of measuring adolescents’ emotion skills with experimental, behavioral, and physiological approaches. Notably, the internal consistency of the distraction subscale of the coping measure was relatively low at .56. Even though this is consistent with previous research using this scale (e.g., [39, 40]), results should be interpreted with some caution. Another limitation of the current study is the fact that each NSSI behavior endorsed could not be disentangled from adolescents’ possible desire to die. Given the study was conducted prior to the newly defined nonsuicidal self-injury disorder in the DSM-5 [41], it was not possible to determine whether adolescents met criteria for this disorder. Future research should incorporate a more nuanced assessment of NSSI and suicide ideation, desire, and attempt. Given the predictive power of NSSI for suicide attempts [2], it would also be worthwhile to examine how coping strategies might impact NSSI specifically in the context of suicidal ideation.

## Conclusions

Taken together, current results contribute to our understanding of how coping strategies may impact NSSI use in youth with emotion expression difficulties. For youth who struggle to identify and express negative emotions, encouraging the use of particular coping strategies (e.g., support seeking, positive reframing) might help to prevent engagement in NSSI in the face of distressing events. These findings are also valuable in providing support for interventions aiming at improving emotion expression skills in adolescents.

## Abbreviations

NSSI: nonsuicidal self-injury
ODD: oppositional defiant disorder
ADHD: attention deficit hyperactivity disorder
DSHI: Deliberate Self-Harm Inventory
EESC: emotion expressivity scale for children
CCSC: Children’s Coping Strategies Checklist
ANOVA: analysis of variance
